# Acceptability, appropriateness and feasibility of the Latin American and Caribbean Code against Cancer: perceptions of decision-makers and health professionals in Argentina

**DOI:** 10.3332/ecancer.2022.1375

**Published:** 2022-04-13

**Authors:** Cecilia Straw, Victoria Sánchez Antelo, Melisa Paolino, Raúl Murillo, Carolina Espina, Silvina Arrossi

**Affiliations:** 1 Faculty of Social Sciences, University of Buenos Aires, Santiago del Estero 1029, Buenos Aires 1075, Argentina; 2 Centre for the Study of State and Society, Sánchez de Bustamante 27, Buenos Aires 1193, Argentina; 3 Centre for the Study of State and Society/National Council for Scientific and Technical Research, Sánchez de Bustamante 27, Buenos Aires 1193, Argentina; 4 San Ignacio University Hospital, Kra 7 40-62, Bogotá, Colombia; 5 International Agency for Research on Cancer, 150 Cours Albert Thomas, 69372 Lyon CEDEX 08, France

**Keywords:** cancer, acceptability, prevention, implementation science, Latin American code against cancer, Argentina

## Abstract

**Background:**

Cancer is an important public health problem. In Latin America and the Caribbean, there were approximately 1,500,000 new cases of cancer and 700,000 deaths due to cancer in 2020. These figures will increase by 78% by 2040 to more than 2.5 million people diagnosed with cancer each year, who will require medical attention, care and support. However, it is estimated that at least 40% of cancers could be prevented by adopting a healthier lifestyle, reducing risky behaviours and implementing recommended health interventions.

**Objective:**

To evaluate the perceptions of health decision-makers and professionals regarding the Latin American and Caribbean Code against Cancer (CLCC) as a support tool for designing and implementing public policies for cancer prevention and control (acceptability, appropriateness and feasibility) in Argentina.

**Methods:**

A qualitative study was conducted using individual, semi-structured interviews with health decision-makers and professionals (*n* = 30). The questions and thematic analysis of the information gathered have been guided by the principles of the Consolidated Framework for Implementation Research: intervention characteristics, outer setting, inner setting and characteristics of individuals.

**Results:**

Health professionals and health decision-makers broadly accepted the proposal of the CLCC as a tool for supporting the design and implementation of public policies for cancer prevention and control, and considered it to be appropriate. Additionally, from the interviewees’ perspective, factors should be ensured to guarantee the implementation of the CLCC as a viable public health policy. They also felt it was right to take the CECC as a model and to adapt its content to the specific characteristics of the Latin American population, customs, lifestyle habits, epidemiological characteristics and, in particular, the Argentinian socio-economic context. Interviewees perceived the CLCC as a health intervention whose complexity varied depending on the recommendation, although most of them were feasible. The broad consensus among the interviewees was that the development of the CLCC could yield numerous advantages in improving cancer prevention and control policy, and responding to the needs of the population. It was also considered to be an opportunity to introduce fundamental changes. With regard to the implementation of the CLCC, interviewees reported a favourable institutional climate, since they perceived that it would receive a priority equal to or greater than the ongoing prevention measures, and that it would have the commitment of the health authorities. They also felt that the implementation of the CLCC in their work environment would not be very complicated, and that the decision-makers and professionals had the necessary capacity to implement it. Finally, they felt that the implementation would be facilitated by the participation and consensus of health decision-makers at the primary care level, and negotiation with industrial and environmental sectors.

**Conclusions:**

Our study shows that health professionals and decision-makers consider the CLCC to be highly acceptable, appropriate and feasible. This would facilitate its implementation as a tool that could enhance current cancer prevention and control policies in Argentina. The results of the study indicate the necessity for the CLCC to be adapted to the socio-economic context of Argentina, and highlight that population adherence to the CLCC recommendations will depend on complex and diverse factors, especially those involving changing unhealthy behaviours linked to cancer risk.

## Background/Introduction

Cancer is an important public health problem. In Latin America and the Caribbean (LAC), in 2020 [1], there were approximately 1,500,000 new cases and 700,000 deaths. Most were cases of prostate, breast and colorectal cancer. Furthermore, the risk of people below the age of 75 developing or dying of cancer is estimated at 18.9% and 9%, respectively. These figures will increase by 78% by 2040 to more than 2.5 million people diagnosed with cancer each year, who will require medical attention, care and support [2]. However, it is estimated that at least 40% of cancers could be prevented by adopting a healthier lifestyle, reducing risky behaviours and implementing recommended health interventions [3].

The European Code against Cancer (CECC for its initials in Spanish), the first edition of which was published in 1987, is a European Commission initiative that seeks to inform people of ways to reduce their risk of cancer [4]. The current fourth edition consists of 12 recommendations, supported by scientific evidence, of behaviours and actions to reduce the risk of illness and death due to cancer. The recommendations were compiled by independent experts, coordinated by the International Agency for Research on Cancer (IARC-WHO) with scientific and technical support from senior researchers in cancer prevention and control in Europe.

The extension of such recommendations to LAC would provide evidence-based recommendations as key cancer prevention tools, to inform people and guide health education in order to promote healthy behaviours and support the development of public policies [5]. This involves gathering, analysing and evaluating scientific evidence to support cancer prevention recommendations which are appropriate to the LAC setting. To develop the Latin American and Caribbean Code against Cancer (CLCC for its initials in Spanish) adapted to the epidemiological, socio-economic and cultural situation of the region, an engagement strategy has been set out for a wide range of regional and international institutions and networks working in the field of cancer prevention and control, where the actors involved take ownership of the future CLCC and become its advocates [5]. To this end, a coalition has been formed that includes the Pan-American Health Organisation and the IARC-WHO, a scientific committee of senior researchers with extensive experience in the region, and an advocacy group representing important LAC organisations such as the Latin American and Caribbean Society of Medical Oncology, the Latin America Cancer Institutes’ Network, the Healthy Caribbean Coalition and the Association of Latin American Leagues against Cancer [5].

In particular, knowing and understanding the perceptions and preferences of health decision-makers and health professionals are essential for deciding the implementation of an innovation such as the CLCC. The specific knowledge and functions of these actors, linked to cancer prevention policies and patients, make them key informants for the implementation of the code in the real world [6].

In order to inform the future development of the CLCC, a multi-centre study took place in Argentina and Colombia to analyse the perspectives of health policy and decision-makers, health professionals and the general population. The study was delayed in Colombia due to the COVID-19 pandemic. It is currently in the final fieldwork stage and its results will be available soon. In this article, we present the results of the study of healthcare professionals and healthcare decision-makers’ perceptions of the acceptability, feasibility and appropriateness of the CLCC as a tool for supporting the design and implementation of public policies for cancer control and prevention in Argentina. In Argentina [2], approximately 140,000 new cases and 70,000 deaths are diagnosed each year, with breast, colorectal and lung cancers having the highest incidence and mortality rates. The fourth National Survey of Risk Factors carried out in 2018 showed that the incorporation of healthy behaviours included in the main recommendations of the European Code (e.g., average daily consumption of fruits and vegetables, limiting daily alcohol consumption, physical activity and control of tobacco use) by the population had been insufficient [7].

## Methods

### Study design

A qualitative study was conducted using semi-structured individual interviews. The formulation of this study was led by the research team and was guided by the dimensions of the Consolidated Framework for Implementation Research (CFIR). The CFIR was developed by US researchers in the late 1990s as part of an initiative to improve the quality of healthcare for veterans [8]. Since then, the constructs have been expanded and are currently being used for designing, implementing and evaluating various healthcare strategies in different contexts and countries. The CFIR provides a menu of theoretical and multidisciplinary constructs that, in the case of formative research, allow for clear and consistent articulation of factors that may potentially affect the results of implementation [9, 10]. In our study, the CFIR guided the design of the interview guides and the result analysis. CFIR constructs were used as predictors [11] of the CLCC implementation. We also incorporated into the analysis the dimensions of acceptability, appropriateness and feasibility proposed by Proctor [6] as follows:

We define acceptability as a set of perceptions of the actors involved regarding whether an innovation or its content is pleasant, desirable or satisfactory.

We define appropriateness as a set of perceptions regarding the fit, relevance or compatibility of an innovation or evidence-based practice from a different context and/or the fit perceived by the actors involved with respect to the fact that an innovation can target or bring attention to a particular issue or problem in a defined context.

We define feasibility as perceptions of the extent to which an innovation can be successfully used or carried out within a given agency or context.

We describe this articulation of theoretical frameworks in Table 1.

### Study location

Argentina is a federal republic made up of 24 provinces, each of which is an autonomous entity responsible for the managing and funding of its own healthcare system. In 2010, the estimated population was around 40 million people [13]. In 2021, 90% of the population lived in urban centres, and 42% were poor [14]. The public health system is free for people without social security (informal workers and their families); people with formal jobs contribute to the funding of healthcare institutions linked to professional activity and private health companies. The Metropolitan Area of Buenos Aires (AMBA for its initials in Spanish) is the largest metropolitan area in the country; it includes the Autonomous City of Buenos Aires and 40 municipalities of the Buenos Aires province. In total, according to the 2010 census, it has almost 15 million residents, who represent 37% of the population of Argentina [13].

### Target population and sample

Purposive sampling was used: 15 healthcare decision-makers involved in management and/or design of policies for prevention and control of cancer risk factors in Argentina, with institutional responsibilities at the national, provincial and municipal levels, as well as 15 healthcare professionals working at first-level care centres from the public, private and social security health sector of the AMBA were interviewed. There was no a *priori* decision about quotas for the distribution of sample member characteristics. The sample was chosen for convenience and ended up being made up of those who accepted the invitation to participate (a male healthcare decision-maker refused to participate and was replaced by a female decision-maker from another province; a male healthcare professional refused to participate and was replaced by a female health professional from another healthcare facility).

### Inclusion and exclusion criteria

Healthcare decision-makers should be state officials in charge of the design and management of policies aimed at cancer prevention at the national, provincial or municipal level. Those healthcare decision-makers who performed healthcare functions at the primary care level were excluded.

Healthcare professionals should have clinical or general medical training, and provide health services for patients at first-level care health centres of AMBA. Those health professionals who were involved in managing cancer control and prevention policies were excluded.

### Data collection instruments

The semi-structured individual interviews were conducted using an interview guide based on dimensions and constructs of the CFIR. Interviews were conducted face-to-face or by phone and lasted 1 hour on average. To make the interviews more dynamic, the interviewees were emailed a card with information about CECC, as well as a link to the CECC website ahead of time so that they could access additional information (https://cancer-code-europe.iarc.fr/index.php/es/).

### Data collection

The data was collected by a researcher trained in social sciences applied to the health sector, between December 2019 and March 2020.

### Analysis technique

The compiled data was analysed by theme [15]. Once the transcription had been completed, all interviews were entered into the ATLAS.ti software (version 7.5.4; ATLAS.ti *Scientific Software Development GmbH*, Berlin). The coding and analysis was done in accordance with four of the CFIR domains and their constructs [9]. Throughout the analysis, the perspectives of professionals and decision-makers were compared. Where agreements and differences were found, these are indicated.

The analysis was carried out by two researchers. The results of the investigation were discussed in joint sessions of the research team, with any disagreement being resolved through a debate.

### Ethical aspects and participant consent

The research protocol was approved by the Centro de Educación Médica e Investigaciones Clínicas “Norberto Quirno” (CEMIC for its initials in Spanish). Before the interview, each participant was explained the objectives of the study and asked to give written informed consent, in order to log the interview and use the answers for analysis. The confidentiality and anonymity of participants was guaranteed.

## Results

### Study participants

Of the 15 health decision-makers, 11 were women and 4 were men, distributed according to the level at which they perform their duties, in the following manner: 5 nationwide, 9 at the provincial level and 1 at the municipal level. The length of time spent in their role was on average 3 years, with a minimum of 6 months and a maximum of 10 years. Of the 15 health professionals, 11 were women and 4 were men; 11 worked in the public health sector, 3 in the private health sector and 1 in the social security sector. The length of time spent in their role was on average 9 years, with a minimum of 1 year and a maximum of 25 years.

### Prior knowledge of the CECC

Health decision-makers and professionals’ prior knowledge of the CECC was very limited, with most being unaware of it.

‘I didn’t know [about the CECC], and it caught my attention, because I was doing rotations for four months in a primary care team in Barcelona, and I was never introduced to it’ (Professional 5, Public, AMBA).

‘Honestly, we already follow these recommendations, since they’re the ones that you’d normally use, but I didn’t have the code [CECC] until you sent me the materials’ (Decision-maker 8, Nationwide).

### Acceptability, viability and pertinence of an CLCC

The proposal of an CLCC was widely accepted by both health professionals and health decision-makers, who also saw it as pertinent. Additionally, from the perspective of those interviewed, in order to ensure the viability of the CLCC being implemented as part of public health policy, within the framework of the Argentinian healthcare system and existing cancer control policies, certain factors will have to be ensured. We present the different dimensions used to analyse acceptability, viability and pertinence below.

### Perceptions regarding an CLCC based on the CECC

The health professionals and decision-makers regarded the CECC as a legitimate model upon which to base the CLCC. Those interviewed saw the prior experience of the CECC as setting a positive precedent, which would have a beneficial effect on the local implementation for both health authorities and the population itself. In particular, among health decision-makers, the prevailing perception was that the very implementation of the CECC in Europe demonstrated the effectiveness of its 12 recommendations.

Despite agreeing on the positive precedent set by the CECC some decision-makers reported that the CLCC would be a different code, adapted to the national reality, to the population’s cultural and epidemiological characteristics and local health systems. As such, this group took the view that the particularities of the CLCC, and the changes necessary to implementing it locally, would dilute any possible influence the code’s European origin could have. Here, there would be a marked difference between local implementation and the code’s European precursor.

‘For me, it is positive to look at where it’s being used and where it’s working. And it’s always good to say “someone has already applied it”’ (Professional 7, Social work, Autonomous City of Buenos Aires).

‘I think that for us to be adapting a code to Latin America that is already being implemented in Europe acts as a good endorsement. It would give people more reassurance’ (Decision-maker 1, Provincial level).

‘Sometimes, the implementation is so complex that the fact that it’s from Europe or the US is anecdotal. Saying “we’re going to create a Latin-American code” changes it all. You can adapt a tool for which the scientific content is similar, but this adaptation changes it when you’re considering all the relevant aspects for its implementation, both in terms of the healthcare system and the community’ (Decision-maker 8, Nationwide).

Both health professionals and decision-makers were in unanimous agreement on the need for the local implementation of the CLCC to be led by the Ministry of Health of Argentina, so as to guarantee a high rate of acceptance among the various actors involved. In addition, some decision-makers expressed nuances regarding the role of the Ministry of Health, emphasising that the leadership role for the CLCC corresponded to the National Cancer Institute. In any case, emphasis was also placed on the need for the leadership of a national institution to be shared among provincial state agencies (e.g., ministries of health, cancer control institutes/agencies).

‘I think that those leading would have to be the Ministry of Health, National Cancer Institute, Provincial Ministry of Health and Provincial Cancer Institute. It’s a line that should not be interrupted’ (Decision-maker 5, Provincial level).

When it comes to the adaptability of specific recommendations, both health decision-makers and professionals were in agreement with the fact that those related to protection from carcinogenic substances in the workplace, and to high radon levels, were not within the realm of individual action in the absence of pertinent public policies. In addition, they saw it necessary to remove the recommendation on hormone replacement therapy, as it is not compatible with current guidelines on cancer prevention.

‘There are two that I found horrible. I have to find out if there’s radiation in my house … I could never tell somebody to do that, they’d kick them out. This can’t be somebody’s job to figure out. Or in the case of the workplace: “protect yourself from carcinogenic substances”. There are loads of places here that don’t comply with that. And if that’s the only place you’re able to find work, are you going to bring that up with the company? That’s where the fear of being fired comes in. The responsibility for this must not fall at the feet of people who, in the case of the workplace, additionally find themselves in an asymmetrical dynamic with the person to which the complaint has to be made. I’m telling you that they’d fire them’ (Professional 2, Public, AMBA).

‘I am entirely unaware of any municipal policy regulating radon levels, or any other natural component’ (Professional 10, Public, AMBA).

‘There are two points that appear irrelevant to me. Hormone replacement therapy, we never talk about that here. I don’t know whether that’s because it has not been proven comprehensively or due to a lack of familiarity’ (Decision-maker 6, Provincial level).

With regard to the need to adapt those CECC recommendations that were accepted, there were differences in the answers depending on the category of the interviewee. Among healthcare professionals, the majority considered it unnecessary to make adaptations. In their opinion, the CECC covers the prevalent types of cancers comprehensively, incorporating their link to behaviours that are not generally associated, by health professionals or the general population, with cancer prevention or risk factor reduction (e.g., alcohol consumption, diet and breastfeeding) as a distinguishing feature. Meanwhile, the decision-makers proposed adapting some recommendations to the Argentinian socio-economic context (e.g., poverty levels, economic activities and the use of agrochemicals), and incorporating new content, such as the human papillomavirus (HPV) vaccine for boys, in order to align it with Argentinian legislation. For them, it would be possible to implement the CLCC if these proposed adaptations were taken into consideration.

‘The content linking cancer prevention matters with alcoholism is very interesting. When educating people on the negative effects of alcohol, I never talk about cancer, and when I talk about diet, I do it with more with chronic diseases in mind, but not cancer. And with breastfeeding, you think about the baby growing well, not about breast cancer. The way that cancer is identified here is good, more light needs to be shed on the causality between these activities and cancer prevention’ (Professional 12, Public, Autonomous City of Buenos Aires).

‘I think that when it comes to the “eat healthily” part, this needs to be adapted. First you need to eat. What does this person have that they can eat? We need to start by looking at the socioeconomic context that this woman, this family, this population finds themselves in. I think that the idea of adapting something European to the Latin-American context is something very important, that we can’t not look at, nor can we fail to consider when making the recommendation itself’ (Decision-maker 8, Nationwide).

Among those interviewed, the perceived complexity of the CLCC as a health intervention varied depending on the recommendation. For healthcare professionals and decision-makers, most of the recommendations were viable (e.g., vaccination, breastfeeding, preventive screening for breast, cervical and colorectal cancer, quitting smoking and smoke-free spaces), as they were aligned with existing policies.

‘With the majority, there is no contradiction found, nor are there overlapping areas, or areas that run counter to what we are trying to develop...’ (Decision-maker 2, Provincial level).

On the other hand, both stressed that to carry out some recommendations (e.g., healthy eating, daily exercise and protection from carcinogenic substances in the workplace) would involve a multiplicity of factors, making their implementation more complicated. For example, the need for a dietary policy facilitating access to healthy foods was mentioned, in addition to the need to design an effective intervention for the changing of unhealthy habits.

‘It always ends up being the case that, for something to bear fruit, the healthcare system can only do so much, as it also depends on social determinants, nutritional policies encouraging healthy eating, fruit and vegetable consumption and alcohol consumption. These policies are often beyond the Ministry of Health. And this isn’t due to a lack of knowledge of which foods improve one’s health, or the protective factors.

This is often not about choice, but rather a question of what people are able, or can afford to make. There is also a cultural element, and often questions around basic needs not being met. We always tend to say: eat healthy, eat fruits and vegetables; and perhaps a change in approach when it comes to nutritional health policy is the most difficult thing’ (Decision-maker 3, Provincial level).

‘It’s very difficult for the people I attend to, to have a healthy diet, because it’s not accessible for them. They eat very fatty things, with lots of sugar and lots of refined flours, because many of these foods are cheaper. It’s difficult to incorporate fruit, vegetables and wholegrains that are more expensive. And for the majority of the people that I attend to, there is a complete lack of health and safety measures in their working conditions. That’s not easy to change, and it’s beyond the remit of a doctor’ (Professional 10, Public, AMBA).

Moreover, the decision-makers mentioned possible opposition from ecological groups to the CLCC, if it were to be developed without a recommendation prohibiting the use of agrochemicals. This is in addition to its rejection by groups whose economic interests could be affected by the recommendations pertaining to healthy behaviour (e.g., tobacco industry, ultra-processed food industry etc.). Another argument from those interviewed, lending credence to the idea of the CLCC’s implementation being highly complex, was possible opposition from the authorities at the primary care level, if the CLCC were to be implemented without a participation and consensus-based strategy, with those actors that must implement it.

‘I think that there will be many groups saying that the CLAC isn’t true. Ecological groups who will say it’s a falsehood, because we don’t have agrochemicals here, and [for them] the only things that lead to cancer. [...] The same thing is happening with the law on agrochemicals, where there’s been lots of toing and froing. A political decision-maker has to know how to navigate these issues, because these groups are very capable of making themselves seen in the media, regardless of how much scientific endorsement this has’ (Decision-maker 5, Provincial level).

‘Everything is complex, because reality is complex. And nobody can be in disagreement with a Code that has national and international endorsement. Although you’ll always have some enlightened person arguing with you about the earth being round. Along with the economic interests at play with some of the things you want to apply’ (Decision-maker 7, National level).

‘There’s always differences in terms of criteria, in a fragmented healthcare system that will respond depending on the level of responsibility [national, provincial or municipal]. I think that for this to not simply be some code printed on a website, or available in a document, all the providers at the primary care level must be involved in working on its introduction’ (Decision-maker 8, Nationwide).

Moreover, both the groups of interviewees mentioned the need to accompany the CLCC with strategic planning, media campaigns, a trained and motivated health staff, longer medical consultations, along with public policies and regulations, and the involvement of non-healthcare institutions in its implementation (e.g., schools).

‘It will be necessary to have a clear objective and strategic planning on the actions involved, and to persevere with these. Each decision-maker has to be certain about what they want to achieve, without losing sight of their objective, and remaining steadfast. It’s often costly, and we get brought down by a budget, a change in management, or by necessity, as in the case of coronavirus now’ (Decision-maker 12, Provincial level).

‘To begin with, it will be necessary for the consultations to be longer, because it takes time to provide counselling, especially if it’s on many topics, and appointments last ten minutes. It’s very difficult to provide both medical attention and counselling in that amount of time. And at the political level of human resources, there should be more staff for primary care’ (Professional 14, Private, Autonomous City of Buenos Aires).

### Implementation of the CLAC in relation to policies for cancer prevention and control

There was a wide consensus among the healthcare professionals that developing a CLAC could bring multiple advantages with respect to the current situation. Both groups of interviewees agreed that the CECC’s recommendations would strengthen actions already under development for the prevention and control of cancer risk factors. They were also of the opinion that the CLCC would mean having recommendations for reducing cancer risks specific to the Latin American context. In parallel, the interviewees emphasised that the CLCC would favour the implementation of changes considered fundamental to ensuring that the policies, programmes and measures implemented have the expected results (e.g., prioritise cancer as a national public policy, assign the necessary budget, develop preventive measures for tumours that are not prioritised and develop measures to promote and communicate preventive behaviours among the population). To complement this, the interviewees mentioned the need to strengthen first-level healthcare, invest in human resources and create regulations and standards to promote healthful behaviours. In their view, implementation of the CLCC could facilitate these changes.

‘Recommendations one and two refer to the policy to reduce tobacco consumption, which does not have the relevance one would like. [There is already work being done to promote] healthy weight and exercise, and it’s possible that, with inter-institutional cooperation, the Code could improve [prevention], along with healthy eating habits. Nothing is being developed at a provincial level with regard to alcohol consumption; very little is being done. We are developing [initiatives] regarding vaccines, breastfeeding and participation in screening programmes, and the Code could strengthen these even further’ (Decision-maker 3, Provincial level).

‘In terms of risk factors, not just for cancer, for me the basic point really is to have an obligatory regulatory framework. I think that regulatory measures should be modified for many of the things proposed in the code. I don’t see a big gap between what this code proposes and the guidelines we have already set for these actions’ (Decision-maker 6, Provincial level).

‘We need to continue in the direction we’re going, but more than change what we need is investment. The impetus is lacking because we don’t have enough investment in human resources, in training for developing the human resources to provide true quality work’ (Decision-maker 13, National level).

Nevertheless, the interviewees agreed that the creation of the CLCC would respond to the needs of the population by providing consistent guidelines for preventing and controlling cancer risk factors. In their opinion, this advantage would represent an opportunity to integrate the actions of different health subsystems (public, private and social security sectors), government jurisdictions (national, provincial and municipal) and civil society organisations that focus on cancer. At the same time, the interviewees underscored the need for collaboration between government ministries (e.g., health, education, industry etc.) and the incorporation of the different sectors and actors involved (e.g. the industrial sector, political representatives, labour unions etc.) in the implementation of the CLCC.

‘Formalising something – in this case, the Code – makes it easier and simpler for people by providing them with information about what we can do. It also means there aren’t so many professional opinions being thrown around. The idea is for everyone to work with the same guidelines for cancer prevention’ (Professional 1, Public, AMBA).

‘Ministries of health, scientific associations, health authorities should take it as a macro approach. From the national level, it should be channelled downward to provide the same working guidelines at the federal, provincial and municipal levels’ (Decision-maker 9, Provincial level).

‘I think that the health and legislative authorities in every province should sit down with the industrial sectors to address this issues more concretely because their input is valuable and, when given the opportunity, their participation is adequate’ (Decision-maker 4, Provincial level).

In particular, health professionals stressed that, to respond to the needs of the population and be implemented as public policy, the CLCC must be an ‘obligatory’ topic at medical appointments. In addition to addressing it within the health system, they added, efforts must continue to help professionals across the national territory understand and adapt the CLCC to real living conditions while working to establish the system in the long term. The professionals interviewed also recommended including community health initiatives to educate the public about healthy habits.

‘I think we can launch awareness campaigns to help health centres that manage primary care: about the age for prevention, because the patients don’t come, or they come late, they’re afraid, or the woman stays home and doesn’t follow the controls she is supposed to. We can do this as healthcare managers or the preventive team’ (Decision-maker 15, Provincial level).

Most of the interviewees believed that healthcare institutions, especially those in the public subsector, would support the implementation of the CLCC and give it the same or more priority as existing cancer prevention policies. There was consensus that the CLCC would help strengthen and deepen some existing preventive measures and underscore the value of community-level work. A majority of interviewees also considered that the health authorities would back the CLCC’s implementation. Their reasoning was that most of its recommendations are consistent with current public policies.

‘It [the CLCC] would allow us to present the actions we are already taking as a comprehensive strategy for prevention of all types of cancers. Our efforts are focused on screening and on trying to reach everyone who has a right to it. [...] If screening is a priority in the Latin American Code - and it encompasses much more than that - then we will be able to implement it as a priority at the ministerial level. My efforts as part of this team will focus on that’ (Decision-maker 3, Provincial level).

‘The code helps me reassess if more interventions are needed, so I would give greater to priority to existing gaps, such as alcohol and sun care among adults, or workplace cancers’ (Professional 5, Public, AMBA).

‘I think it would be very well received by provincial health authorities and governments. Intermediate associations and NGOs [non-governmental organisations] would accept it immediately’ (Decision-maker 1, Provincial level).

‘I don’t think the health authorities would object, because some of these things are already being done’ (Professional 7, Labour union, CBA).

The few decision-makers who maintained that the CLCC would be given lower priority than existing initiatives argued that its contents would exceed the scope of the cancer prevention measures they can implement.

‘The top priorities are healthy eating habits and physical activity. [...] The rest of the Code is less of a priority’ (Decision-maker 6, Provincial level).

### Perceptions of capacities for implementing the CLCC as a health intervention

The perception of health professionals and decision-makers was that implementing the CLCC as a health intervention in the context of their work (e.g., for health professionals, their medical practices; for decision-makers, public policy design and implementation) would be of low complexity. In their opinion, most of the CLCC’s recommendations are consistent with existing policy guidelines for preventing and controlling cancer risk factors. At the same time, some decision-makers also highlighted that the availability of human resources at the first level of care, especially those who work at the territorial level (e.g., health promoters and healthcare agencies) would facilitate implementation in health services. On the other hand, they considered that the protocolisation of the CLCC would function as a reminder of the topics to be addressed in the medical consultation. Likewise, both decision-makers and health professionals stated that clarity in the wording of the recommendations would facilitate the communication of the CLCC’s contents.

‘I think it could be easily implemented because we are working in line with the recommendations so it would be part of the implementation of many policies. One more aspect, so it would not be difficult’ (Decision-maker 14, National level).

‘I think it would be easy during the consultation because these are more or less the majority of topics we usually work with’ (Professional 2, Public, AMBA).

‘What facilitates implementation is the human resource. The availability of promoters, health workers and interdisciplinary teams facilitates it’ (Decision-maker 9, Provincial level).

In line with the perception of self-efficacy to implement the intervention, both types of interviewees reported that they, as well as their colleagues, had the necessary capabilities to incorporate the CLCC as a cancer prevention strategy. For health professionals, this positive perception of self-efficacy was linked to their general medical training, which would facilitate the approach of health behaviours promoted by the Central European Cancer Centre (CECC) within the framework of the medical consultation.

‘It would be easy for me because of the general training I have and not being focused on some very particular aspect of treating a disease’ (Professional 3, Public, AMBA).

## Discussion

Our study is the first formative research to evaluate the acceptability, appropriateness and feasibility of the CLCC as a tool to support the design and implementation of public policies for cancer prevention, and control from the perspective of decision-makers and health professionals in Latin America. The study design was based on the CFIR, which is a conceptual framework of implementation science that is especially suitable to identify, in the pre-implementation stage of a health intervention, the necessary adaptations to the local context. The results show that the implementation of the CLCC has a high acceptability by health professionals and decision-makers, who in turn perceive it as relevant. Additionally, the study identified those factors that, from the perspective of the actors interviewed, should be ensured to guarantee the viability of the implementation of the CLCC as a public health policy.

Our results showed very little prior knowledge about the existence of the CECC by the interviewees. However, during the interview, once they were informed about the European experience, there was a high acceptability about the elaboration of the CLCC based on the CECC. This is a key finding of our study since the legitimacy of the source or origin of the intervention is associated with the success of its implementation [16].

Likewise, the interviewees considered that the availability of the CLCC would bring multiple advantages relative to the current situation, mainly by providing specific recommendations to reduce the risk of cancer in the country and Latin America, and have the possibility to strengthen existing public policies for the prevention and control of cancer risk factors, and to introduce the changes that these policies need. This perception of interviewees coincides with the results of the National Survey of Risk Factors (2018) that showed that policies implemented in recent years to promote healthy consumption and practices (reducing consumption of trans fats and sodium [7, 17], reducing tobacco consumption [7, 17] and promoting physical and sporting activity [7, 17]) had mixed results, limitations in the application and controls of rules and regulations and vacancy areas [7] on which it is a priority to implement new interventions that contribute to the prevention and reduction of cancer risk. The recognition that an intervention has relative advantages over the current situation has been considered as a key factor in the success of a health intervention [18]. In a study evaluating a weight control programme in the US, health decision-makers identified the relative advantages of the programme (e.g., boosting the existing programme and expanding it and incorporating an interdisciplinary perspective into the weight control programme) as the only positive factor linked to its successful implementation [19].

In Argentina, cancer is one of the main causes of illness and death. Our study showed that the implementation of the CLCC is perceived as pertinent, i.e., it would allow channeling or solving a priority health problem. Indeed, both decision-makers and professionals reported that the CLCC would respond to the needs of the population in terms of cancer prevention by facilitating the unification of the guidelines of these policies in the different health subsystems. For those interviewed, the fragmentation of the health system and, at times, the contradiction of clinical practices and health policies implemented in the health subsystems and in the different jurisdictional levels (national–provincial–municipal) generate inequity in access to evidence-based practices. Various studies have documented that in Argentina the inequity in the distribution of health coverage and access to services is alarming, and that the difficulties in ensuring that the system covers the needs of the entire population have had more to do with the lack of policy coordination than with the absence of policies [20, 21]. In this sense, the CLCC would provide a unifying framework in order to guarantee that the entire population has the same possibility of receiving scientifically validated and high-quality practices and interventions for the prevention and control of cancer.

In our study, the high viability of the CLCC was linked to how respondents perceived its implementation in relation to four characteristics considered key to the success of a health intervention [18, 22, 23]: a). It would constitute a relative advantage in relation to the current public policy situation; b). It would receive a higher priority from the health authorities; c). Its implementation as an intervention to be applied in the medical practice would be relatively low complexity; and d). It would be adapted to the care practices of health professionals.

The perception of high self-efficacy to incorporate the CLCC as an intervention in the professional fields (medical practice and public policy management) is a key factor for the feasibility of its implementation. Evidence shows that perceptions of simplicity to incorporate an intervention in labour practice by stakeholders have a positive association with effective implementation [18, 22]. A study conducted in Vietnam showed that an intervention to increase the adoption of tobacco use treatment guidelines [24] had low acceptability and perceived feasibility by health professionals. In this study, the main problems identified by the interviewees were the greater complexity of the intervention compared to current practices, its incompatibility with overload and workflows of clinical practice and competition with other priorities assigned to chronic non-communicable disease prevention programmes by health authorities. In our study, although the need to adapt the timing of the medical consultation to incorporate the CLCC as a health intervention was also mentioned, the perception of high self-efficacy prevailed. This is linked to two key aspects: the majority of the recommendations coincide with current policy guidelines and human resources for the first level of care have the specific training to implement CLCC in the framework of medical consultation.

The acceptability, appropriateness and viability of the CLCC are likewise linked with a positive perception on the part of those interviewed with regard to the health authorities’ commitment to its implementation. Various studies [19, 25, 26] have shown that the participation and commitment of the authorities are fundamental for communicating objectives, establishing and monitoring goals and facilitating feedback among the team members involved. Thus, the commitment from the health authorities creates a climate of favourable implementation which, at the same time, facilitates effective implementation [18].

In our study, those interviewed identified factors that would need to be assured in order to guarantee the viability of the CLCC’s implementation as a public health policy. To that end, they emphasised the necessity for participation and consensus among decision-makers in the area of first-level healthcare, and negotiation with economic groups (e.g., the food industry) and ecologists. These findings are relevant as the need to build consensus and strengthen governing processes is considered a fundamental factor for changing or introducing new health interventions [27]. In a study that analysed the factors linked to the effective introduction of cervical screening with HPV tests [27], it was shown that the participation of health system authorities and heads of different health services at a local level from the first phases of the project was key in guaranteeing that the intervention would have the desired results with respect to the screening, diagnosis and treatment of pre-cancerous lesions.

Our results showed that health professionals reacted positively to the CLCC including recommendations related to food and consumption of alcoholic beverages, as they consider that, in general, there is a limited link between these issues and cancer prevention, among their peers as well as the population. However, those interviewed raised questions as to the feasibility of recommendations involving changing eating habits and unhealthy behaviours. Both the health professionals as well as decision-makers interviewed reported that the recommendations related to healthy eating, consumption of alcohol, body weight and daily exercise would be the most difficult with regard to compliance due to the many factors that intervene in changing people’s behaviours. On the other hand, those interviewed underlined the necessity of improving socio-economic conditions so these recommendations could be incorporated by the general population. This is of fundamental importance due to the deterioration of living conditions suffered by a large portion of the population of Argentina in recent years. In 2021, in Argentina, 12 million people were classified as living in poverty situations and 3 million more were in indigent situations [14]. Furthermore, there has been a sustained increase in the cost of food and non-alcoholic beverages (around 53% between June 2020 and June 2021) [28]. Additionally, during just this last year, a loss of employment positions of 12.3% was noted, which has a different impact depending upon the degree of formality: while employment in registered positions fell by 5%, in non-registered employment the figure was 26.5%, a number that worsened more among women (28%) [29]. This scenario shows that socio-structural conditions can affect compliance with the CLCC’s recommendations with regard to healthy eating or avoiding exposure to carcinogenic substances in the work environment. Our results point out the need to support public policies that facilitate adoption of preventive behaviours and no exposure to cancer risk factors.

The results obtained contribute to a better understanding of the perception of decision-makers involved in prevention policies and professionals working at primary health care. This will contribute to the strengthening of the actions for cancer prevention and reduction of cancer risk in the country.

One limitation of the research is that interviewed health professionals worked in AMBA, which will somewhat limit the transferability of the results. Therefore, further research will be needed to adapt the CLCC to local characteristics, health systems and the needs of the populations in other countries in the region. In any event, the data collected in this study correspond to the major urban conglomerate of Argentina, one of the countries with highest incidence and mortality rates. in Latin America and the Caribbean [2].

## Conclusion

Our study shows a high degree of acceptability, appropriateness and viability of the CLCC on the part of healthcare professionals and decision-makers. This would facilitate its implementation as a tool that could enhance current cancer prevention and control policies in Argentina and the LAC region.

The perception of the CLCC as an intervention that would provide advantages in comparison to the current situation in cancer prevention and control, that would respond to the needs of the population in a setting with trained human resources to incorporate it into primary health care constitutes the basis for the code successful implementation..

Likewise, the results of the study indicate the necessity for the CLCC to be adapted to the socio-economic context of Argentina, and highlight that population adherence to the CLCC recommendations will depend on complex and diverse factors, especially those involving changing unhealthy behaviours linked to cancer risk.

## List of Abbreviations

LAC: Latin America and the Caribbean; CECC: Código Europeo Contra el Cáncer (The European Code Against Cancer); CFIR: Consolidated Framework for Implementation Research; CLCC: Código Latinoamericano contra el Cáncer (Latin American Code Against Cancer); HPV: Human papillomavirus.

## Conflicts of interest

The authors declare no competing interests.

## Funding

This article presents independent research financed by the International Agency of Cancer Research (IARC-WHO) through a subsidy awarded by the American Cancer Society (ACS). The opinions expressed are the authors’ and not necessarily those of these institutions.

## Figures and Tables

**Table 1.** Domains, constructs, CFIR definitions and implementation outcomes.

Domain	Construct		Definition	Implementation outcomes
I. Intervention characteristics	Intervention source		Legitimacy of CECC as an external intervention into the institution to which healthcare professionals and healthcare decision-makers belong	Acceptability
	Relative advantage		Advantage of implementing the CLCC over an alternative strategy	Acceptability
	Adaptability		The degree to which the CLCC can be adapted, refined or redefined to meet local needs	Acceptability Appropriateness Feasibility
	Complexity		Perceived difficulty of the CLCC implementation	Acceptability Feasibility
II. Outer setting	Patient needs and resources		Perception of the degree to which the CLCC to meet patient needs	Acceptability Appropriateness
	Peer pressure		Pressure from any external entity to implement an intervention	Acceptability Feasibility
	External policies and incentives		Strategies to promote interventions through policies and regulations.	Feasibility
III. Inner setting	Implementation climate	Tension for change	Perception of individuals regarding the need to change the current situation	Acceptability Feasibility
		Compatibility	CLCC compatibility with the existing norms, values, workflows and systems	Acceptability Appropriateness Feasibility
		Relative priority	Shared perception of individuals regarding the importance of implementing the CLCC within their organisation	Acceptability Appropriateness Feasibility
	Readiness for implementation	Leadership engagement	Commitment, participation or responsibility of the authorities related to the implementation	Acceptability Appropriateness Feasibility
IV. Characteristics of individuals	Knowledge and beliefs about the intervention		Knowledge and beliefs about or attitudes towards the CLCC	Acceptability
	Self-efficacy and efficacy of colleagues		Capacity of an individual to execute a course of action aimed at achieving the implementation objectives	Appropriateness Feasibility

‘Self-making’ based on the adaptation of Damschroder [9], Proctor [6], Kirk [11] and Meyerson [12].
